# Chromosome‑scale genome assembly of the bovine parasite *Tritrichomonas foetus* using short-read, long‑read and Pore‑C sequencing identifies conserved and lineage‑specific gene families relevant to host interaction

**DOI:** 10.1017/S0031182026102716

**Published:** 2026-08

**Authors:** John Harvey Santos, Antonino Cavallaro, Elizabeth M. Ross, Hannah Siddle, Gry Boe-Hansen, Ali Raza, Loan Nguyen, Ala Tabor

**Affiliations:** 1 Centre for Animal Science, Queensland Alliance for Agriculture and Food Innovation, The University of Queensland, St Lucia, QLD, Australia; 2 School of Veterinary Science, The University of Queensland, Gatton, QLD, Australia; 3 School of Environmental and Rural Science, University of New England, Armidale, NSW, Australia; 4 School of Chemistry & Molecular Biosciences, The University of Queensland, St Lucia, QLD, Australia

**Keywords:** bovine trichomonosis, comparative genomics, orthogroups, Pore-C, structural variants, *Tritrichomonas foetus*

## Abstract

*Tritrichomonas foetus* is the causative agent of bovine trichomonosis, a venereal disease that compromises cattle fertility and causes major economic losses. We report a chromosome-scale genome assembly of *T. foetus* isolate, TfOz5, from Queensland, Australia generated using Oxford Nanopore long-read sequencing and Pore-C chromatin conformation capture. The 137.7 Mb assembly spans 5 chromosomes with an N50 of 24 Mb. Repeat analysis revealed 43.6% repetitive content dominated by DNA transposons. Gene prediction identified 32 104 protein-coding genes of which 98.3% were assigned to orthogroups. Comparative analysis with the KV-1 genome detected 160 structural variants, including deletions (40.6%), insertions (22.5%), duplications (16.3%), inversions (13.1%) and translocations (7.5%). Across 9 *Parabasalia* species, we observed high gene family conservation and 305 species-specific orthogroups in TfOz5. Phylogenetic analysis confirmed the monophyletic cluster of *T. foetus* strains (K, KV-1 and TfOz5). Inter-species comparison with the human urogenital parasite *Trichomonas vaginalis* G3 revealed conserved gene sets between *T. foetus* TfOz5 and *T. vaginalis G3* that were significantly enriched for core cellular functions (translation, cell cycle and mitochondria-related homology hits), while divergent genes highlighted species-specific host interactions. *Trichomonas vaginalis G3* showed enrichment for proteolysis, regulation of DNA-templated transcription and symbiont–host adhesion, traits consistent with persistence in the human genital tract. Similarly, *T. foetus* TfOz5 host interactions align with long-term survival in the bovine reproductive niche. This chromosome-scale assembly provides a valuable genomic framework for investigating parasite evolution, identifying candidate genetic features associated with host adaptation and supporting the development of next-generation recombinant vaccines to control bovine trichomonosis.

## Graphical abstract

**Figure:**
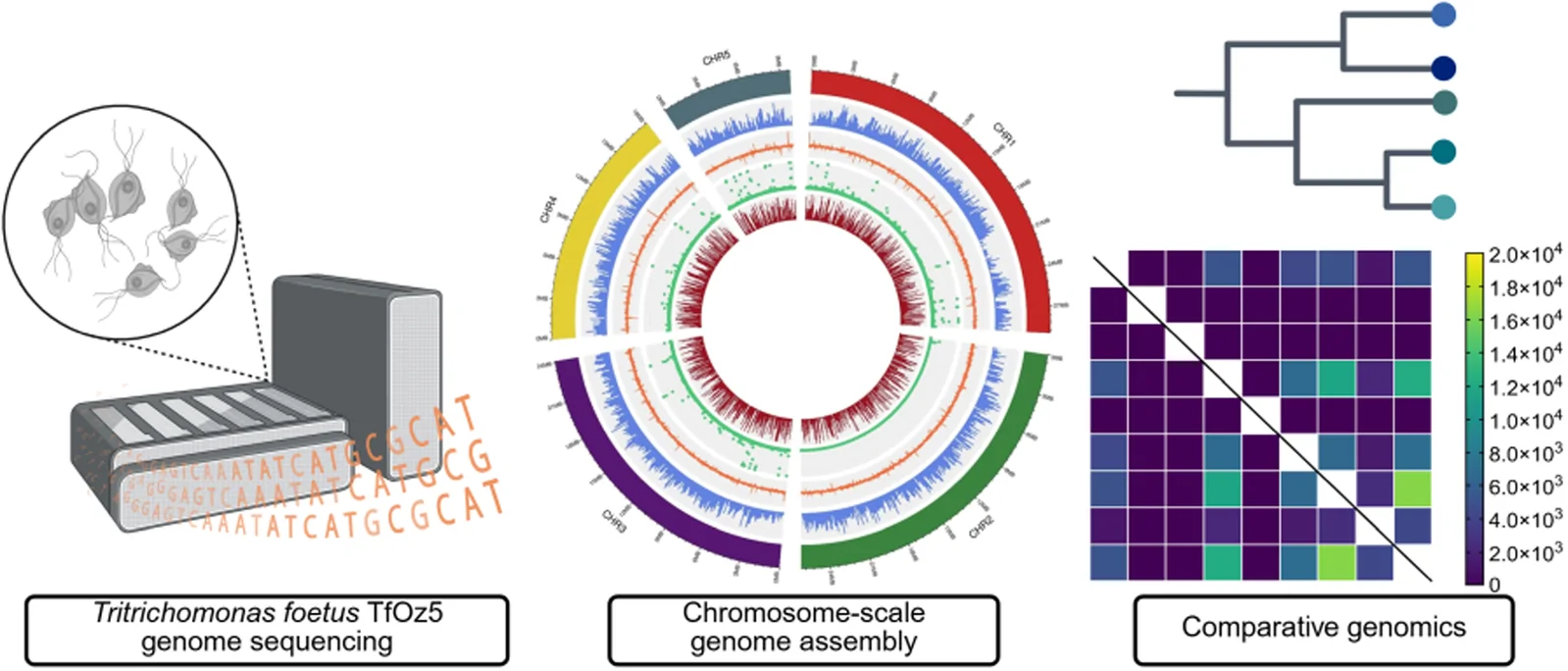

## Introduction

*Tritrichomonas foetus* is a flagellated protozoan parasite and the causative agent of bovine trichomonosis, a venereal disease that occurs worldwide and leads to early embryonic loss, infertility and reproductive failure in cattle (Rae, 1989; BonDurant, 1997). The disease is reported globally, particularly in regions where natural mating predominates, and artificial insemination is not routinely practiced (BonDurant, 1997). Bulls act as asymptomatic carriers, enabling persistent transmission within and between herds (Clark et al., 1984). Globally, bovine trichomonosis imposes significant economic losses due to reduced calving rates, increased culling and costs associated with diagnostics and herd management. For example, in the United States and parts of South America, outbreaks have been linked to substantial reductions in herd productivity (Campero et al., 2003; Rodning et al., 2008; de Oliveira Filho et al., 2018). In Australia, the disease remains endemic in Northern cattle populations, particularly in Queensland and the Northern Territory where prevalence in bulls varies between herds, with prevalences in older bulls of up to 11.4–15.4% (Calvani et al., 2021; Irons et al., 2022). The economic burden is considerable, with studies estimating up to a 35% reduction in return per cow in infected herds, translating to annual losses exceeding AUD $20 million in Northern Australia (Irons et al., 2022). Control efforts are hindered by the logistical challenges of implementing surveillance and reproductive management programmes in remote pastoral regions.

Despite its veterinary importance, genomic resources for *T. foetus* have historically remained limited, with early resources represented by fragmented short-read assemblies such as the K strain draft genome (Benchimol et al., 2017), although chromosome-level resources have recently become available for the KV-1 strain (Abdel-glil et al., 2024). Until recently, the most widely used *T. foetus* genomic resource was the K strain draft genome, isolated from an infected bull in Brazil (Benchimol et al., 2017). This draft genome was assembled using short reads and has 3730 contigs organized into 1573 scaffolds with a genome size of 68.5 Mb (Benchimol et al., 2017). Although *T. foetus* strain K has been instrumental in advancing our understanding of gene content, metabolic pathways and comparative genomics in parasites (Westrop et al., 2017; Dąbrowska et al., 2020; Alonso et al., 2022), its highly fragmented nature has posed significant challenges. In particular, there are limited investigations into chromosomal architecture, structural variation and repetitive regions, features that are essential for elucidating genome evolution and host adaptation.

Recently, a chromosome-scale genome assembly of the *T. foetus* KV-1 strain, isolated from an infected bull in the Czech Republic was published, representing the first *T. foetus* genome assembled at the chromosomal level using long reads (Oxford Nanopore Technologies) and Hi-C scaffolding (Abdel-glil et al., 2024). The *T. foetus* KV-1 assembly spans ∼148.7 Mb, with 5 chromosomes and annotation of 41 341 protein-coding genes, providing a major advance for functional genomics and comparative studies (Abdel-glil et al., 2024). Nevertheless, as with other repeat-rich trichomonad genomes, repeat content and paralogous gene families can complicate genome assembly and automated gene annotation. In particular, highly similar paralogous sequences may be difficult to distinguish computationally, potentially leading to fragmented, duplicated or over-predicted gene models. Although both KV-1 and TfOz5 each represent single isolates, TfOz5 provides an additional chromosome-scale genome from a geographically distinct Australian isolate, complementing the European KV-1 assembly and enabling comparative analysis of genome structure and gene content between regional *T. foetus* isolates. Importantly, KV-1 does not capture variation in Australian isolates, which may harbour unique structural features and antigen repertoires relevant for local vaccine design. Thus, an additional chromosome-scale *T. foetus* genome provides a valuable resource for comparative genomic analysis between geographically distinct isolates and will support future pan-genomic studies as more representative *T. foetus* genomes become available.

Chromatin conformation capture methods such as Hi-C have been widely used to scaffold parasite genomes to chromosome scale (Gupta et al., 2021; Fu et al., 2022; Hassan et al., 2023). Hi-C captures pairwise chromatin interactions using short reads, improving scaffolding but with limited resolution in repetitive or structurally complex regions (Goodwin et al., 2016; Ott et al., 2018). Pore-C, a nanopore-based alternative, sequences long DNA fragments containing multi-way chromatin contacts, enabling detection of higher-order structures and long-range interactions with fewer computational assumptions (Deshpande et al., 2022). Unlike Hi-C, Pore-C improves scaffolding in repetitive regions and preserves native DNA methylation. Recent studies in cattle using Pore-C showed that higher-order chromatin interactions could be captured, with more than 50% of contacts having an order greater than 3, whereas short reads are limited to identifying pairwise contacts (Nguyen et al., 2023). The Brahman genome assembly using Pore-C produced a highly continuous, chromosome-scale reference genome that exceeds the continuity of the current annotated bovine reference assembly (Nguyen Loan et al., 2023). Pore-C has also been applied to scaffold the *Bovicola ovis* reference genome (Ong et al., 2024). In that study, similar levels of interaction capture were obtained using 40% less sequencing data (Ong et al., 2024). These findings highlight Pore-C’s advantages for assembling and interpreting complex genomes rich in repeats and structural variants.

In this study, we generated a high-quality chromosome-scale genome assembly of *T. foetus* TfOz5 isolated from a naturally infected bull in Queensland, Australia using Oxford Nanopore Technologies (ONT) long-read sequencing in combination with Pore-C. Comparative genomics across *Parabasalia* reveals insights into trichomonad evolution linked to host adaptation. This chromosome-scale assembly establishes a robust foundation for molecular epidemiology, comparative genomics and the development recombinant vaccine strategies to control bovine trichomonosis globally.

## Materials and methods

### Tritrichomonas foetus *TfOz5 source and culture*

*Tritrichomonas foetus* isolate TfOz5 used for sequencing was obtained from a cattle property in Queensland, Australia. TfOz5 represents a single purified Queensland field isolate stock and was not generated by pooling or combining multiple isolates. Isolation and purification of the isolate was performed following protocols previously described by Santos et al., (2025). Briefly, TfOz5 was selected from a broader Australian field collection of 66 *T. foetus* qPCR-positive samples collected from 20 cattle herds across Queensland and the Northern Territory, Australia. TfOz5 was chosen for genome sequencing because it had previously been used as the vaccine strain in our Australian whole-cell killed *T. foetus* vaccine studies, including our recent bull vaccination trial, in which the TfOz5 Queensland isolate was used as the vaccine strain (Santos et al., 2026). The isolate also grew reliably in culture, was free from detectable microbial contamination following purification and culture monitoring and yielded sufficient high-quality genomic DNA for short-read, long-read and Pore-C sequencing. Although TfOz5 was not cloned from a single parasite cell, it was derived from 1 field isolate stock rather than a pooled mixture of isolates; therefore, any genetic variation would reflect minor within-isolate variation rather than mixed-isolate contamination. Samples were transported in *Trichomonas foetus* medium (TFM) at 22–37 °C to QAAFI laboratories in Brisbane where they were incubated at 37 °C for 4–5 days. Cultures were then cryopreserved in freezing medium (10% foetal calf serum, 80% RPMI 1640, 10% DMSO) and stored at −80 °C. To optimize growth and purify the cultures, the isolate was maintained in TFM prepared by dissolving 12.5 g/L neutralized liver extract, 5 g/L tryptose and 1.5 g/L Bacto agar in deionized water, adjusting the pH to 7.4 and autoclaving at 121 °C for 15 min. After cooling, the medium was adjusted to pH 7.4, autoclaved and supplemented with 50% heat-inactivated bovine serum and appropriate antibiotics and antimycotics (Table S1). Fungal and yeast contamination was assessed microscopically, while bacterial contamination was monitored using blood agar culture.

To maintain the TfOz5 isolate, TFM was supplemented with antibiotics at the following concentrations: 1040 µg/mL penicillin, 562 µg/mL streptomycin, 1.2 µg/mL amphotericin B and 16 µg/mL gentamicin. For procedures requiring liquid cultures, such as DNA extraction, agar was omitted from the medium to facilitate the recovery of pure *T. foetus* cells. To preserve anaerobic conditions without hindering cell harvest a solid agar plug was placed over the liquid culture medium.

### Genomic DNA extraction

Genomic DNA was extracted from *T. foetus* TfOz5 cultures using cold alkaline lysis method. Cultures were centrifuged at 1000 ***g*** for 10 min at 4 °C, and the resulting pellets were washed twice with 50 mL of ice-cold phosphate-buffered saline (PBS). The washed cell pellets were resuspended in 1 mL of PBS, centrifuged again at 1000 ***g*** for 10 min at 4 °C, and the supernatant was discarded. The dry pellets were stored at −20 °C overnight.

For lysis, 1 mL of lysis buffer (100 mM Tris-HCl, pH 9.0; 100 mM NaCl; 10 mM EDTA; 1.5% SDS) was added to each pellet, and samples were thawed on ice until complete cell lysis was observed. An equal volume (1 mL) of phenol:chloroform:isoamyl alcohol (25:24:1; Merck, Australia) was added, and the mixture was gently inverted to mix. Samples were incubated at 4 °C for 20 min with shaking at 900 rpm, followed by centrifugation at 14 800 ***g*** for 10 min at 4 °C. The aqueous phase was carefully transferred to a new tube. DNA was precipitated by adding 2 volumes of cold 100% ethanol (Thermo Fisher, Australia) and incubating at −20 °C for 30 min. Samples were centrifuged at 14 800 ***g*** for 20 min at 4 °C, the supernatant was discarded, and the pellet was washed with 500 μL of 70% ethanol. After centrifugation at 14 800 ***g*** for 10 min at 4 °C, the supernatant was removed, and the DNA pellet was air-dried for 5 min.

The pellet was resuspended in 50 μL of double-distilled water (ddH₂O) and treated with 50 μg RNase (Thermo Fisher, Australia) overnight at room temperature. DNA was re-precipitated by adding 2 volumes of ethanol and 0.1 volume of 3 M sodium acetate (pH 5.2), followed by incubation at −20 °C for 30 min. Samples were centrifuged at 14 800 ***g*** for 20 min at 4 °C, washed again with 500 μL of 70% ethanol and centrifuged at 14 800 ***g*** for 10 min at 4 °C. The final DNA pellet was air-dried for 5 min and resuspended in 50 μL ddH₂O. DNA quality and concentration were assessed by gel electrophoresis, spectrophotometry and Qubit™ 4 fluorometer (Thermo Fisher, USA). This high-quality genomic DNA was then used for whole-genome sequencing with Oxford Nanopore long reads and Illumina short reads.

### Pore-C DNA extraction

Pore-C DNA extraction was performed as previously described (Nguyen Loan et al., 2023), with minor modifications. Briefly, cells were resuspended in 1 mL of cold phosphate-buffered saline (PBS) and centrifuged at 300 ***g*** for 5 min at 4 °C. The resulting pellet was resuspended in 1% formaldehyde solution and incubated at room temperature for 10 min to allow cross-linking.

For cell lysis, 550 μL of protease inhibitor cocktail–permeabilization solution (10 mM Tris-HCl, pH 8.0; 10 mM NaCl; 0.2% IGEPAL CAS630; and 50 μL protease inhibitors) was added to the cross-linked pellet, followed by incubation on ice for 15 min. The suspension was centrifuged at 500 ***g*** for 10 min at 4 °C, and the supernatant was discarded. The pellet was gently resuspended in pre-chilled 1.5 × digestion reaction buffer. To denature chromatin, 1% SDS was added directly to the suspension, which was then incubated at 65 °C for 10 min at 300 rpm. The sample was cooled on ice, and 10% ECOSURF EH-9 was added to quench the SDS. Chromatin digestion was carried out overnight at 37 °C using 45 units of *NlaIII* (New England BioLabs, USA). The restriction enzyme was inactivated by incubating the sample at 65 °C for 20 min at 300 rpm in a ThermoMixer (Eppendorf, Australia). Proximity ligation was performed by adding the ligation reaction mix and incubating at 16 °C for 6 h at 300 rpm in a ThermoMixer (Eppendorf, Australia).

To reverse cross-links and degrade proteins, a protein degradation solution (5% Tween-20, 0.5% SDS and proteinase K) was added, followed by incubation at 56 °C for 18 h at 300 rpm. The reaction mixture was then cooled to room temperature. DNA was extracted by adding pre-chilled phenol:chloroform:isoamyl alcohol (25:24:1), followed by mixing via inversion and centrifugation at 16 000 ***g*** for 15 min at 15 °C. The aqueous phase was transferred to new tubes. DNA was precipitated by adding 0.2 volumes of 5 M NaCl, 0.1 volumes of 3 M sodium acetate (pH 5.2) and 3 volumes of ice-cold 100% ethanol. Tubes were inverted to mix and incubated at −80 °C for at least 1 h. Samples were centrifuged at 16 000 ***g*** for 30 min at 4 °C. The supernatant was discarded, and the DNA pellet was washed with 70% ethanol. Finally, the DNA was resuspended in Tris–EDTA buffer for downstream applications.

### Library preparation and nanopore sequencing

Crosslinked and genomic DNA were prepared for sequencing using the SQK-LSK110 genomic sequencing kit (Oxford Nanopore Technologies, Oxford, UK), following the manufacturer’s protocol with modifications as described (Lamb et al., 2020). The prepared libraries were sequenced on a PromethION P24 device (Oxford Nanopore Technologies, Oxford, UK) using a FLO-MIN106 (R9.4.1) flow cell for 48 h. Base calling was performed using Guppy v6.5.7 in super-high accuracy mode on a high-performance computing cluster. Raw nanopore reads were filtered and quality-trimmed using NanoFilt v2.8.0 (reads < 20 kilobase pairs (kbp) and *Q* score < 12) (De Coster et al., 2018).

### Illumina library preparation and short-read sequencing

Library preparation and short-read sequencing of *T. foetus* TfOz5 genomic DNA were performed by the Australian Genome Research Facility (AGRF Ltd, Melbourne, VIC, Australia). Extracted genomic DNA was processed using the DNA Prep M library preparation kit (Illumina, USA), and sequencing was conducted on the Illumina NovaSeq X Plus platform, generating 150 bp paired end reads. Image analysis was performed in real time by the NovaSeq Control Software (NCS) v1.2.2.48004 and Real Time Analysis (RTA) v4.6.7, running on the instrument computer. RTA performs real-time base calling on the NovaSeq instrument computer. Then the Illumina DRAGEN BCL Convert 07.031.732.4.3.6 pipeline was used to generate the sequence data. The quality of the raw reads was assessed using FastQC v0.12.0 (Wingett and Andrews, 2018), and adapter trimming and quality filtering were performed using fastp v0.24.1 (default parameters) (Chen et al., 2018).

### De novo genome assembly and quality assessment

Adapter sequences were removed from raw nanopore reads using Porechop v0.2.4 (default parameters) (Wick et al., 2017). Reads shorter than 20 kbp and those with an average Q-score below 12 were excluded from downstream analysis. Genome characteristics, including haploid genome size and heterozygosity, were estimated using GenomeScope 2.0 (Vurture et al., 2017), which analyses the distribution of k-mers in raw sequencing reads without requiring a reference genome. This step provided valuable insights into the genome’s complexity and guided the selection of appropriate assembly parameters. Filtered long reads were assembled *de novo* using Flye v2.9.1 (Kolmogorov et al., 2019). To improve assembly accuracy, the draft genome was polished using Medaka v1.9.1, employing the original long-read data. Four rounds of Medaka polishing were performed to improve the long-read consensus assembly. Further refinement was performed using Polypolish v0.5.0 (Wick and Holt, 2022) with high-quality Illumina short reads. Three rounds of Polypolish were then performed to improve base-level accuracy and correct residual small insertion/deletion and substitution errors. The completeness and quality of the assembled genome were evaluated using QUAST v5.3.0 (Gurevich et al., 2013) and BUSCO v5.4.5 (Seppey et al., 2019) on the eukaryota_odb10 dataset. The polished genome assembly was then used for Pore-C scaffolding. Pore-C reads were not used to correct the DNA sequence itself; rather, they were used to generate chromatin contact information for ordering and orienting scaffolds into chromosome-scale assemblies. Therefore, Pore-C reads were not polished as part of the genome consensus correction process.

### Chromosome assembly of T. foetus TfOz5 using Pore-C

To create a chromosome-scale assembly of the *T. foetus* TfOz5 genome, Pore-C data were processed using the Snakemake Genome analysis pipeline (Köster and Rahmann, 2018) developed by Oxford Nanopore Technologies. Briefly, a virtual *in silico* restriction map was generated using the draft genome and the restriction enzyme *NlaIII*, implemented via the pore_c refgenome virtual-digest tool. Subsequently, Pore-C reads were aligned to the draft genome using BWA v0.7.18 (Li and Durbin, 2009). The resulting alignments were annotated and assigned to their corresponding restriction fragments based on the virtual digest, enabling the reconstruction of multi-way chromatin contact information. To facilitate scaffolding, the multi-way contact data were decomposed into pairwise interactions, which were then used as input for SALSA v2.3 (Ghurye et al., 2017). The final chromosome-level scaffolds were visualized and manually curated using Juicebox v2.5.0 (Robinson et al., 2018).

### Comparative analysis of structural variants between TfOz5 and KV-1

Structural variants (SVs) in the *T. foetus* TfOz5 genome were identified using Sniffles2 v2.6.0 (Smolka et al., 2024), a structural variant caller optimized for long-read sequencing data. Although *T. foetus* strain K is the designated reference genome, its lower assembly level and potential structural fragmentation posed challenges for SV analysis, limiting the resolution and accuracy of comparative genomic insights. To overcome this, we used the chromosome-level assembled KV-1 genome as the reference for SV calling. Long reads from *T. foetus* TfOz5 were aligned to the KV-1 reference genome using minimap2 v2.27 (Li, 2018). Alignment files were processed with samtools v1.21 (Danecek et al., 2021) to generate sorted and indexed BAM files. Sniffles2 was then applied to the alignments with a minimum read support threshold of 10 to ensure detection of high-confidence SVs. To further confirm the reliability of the identified SVs, we performed validation using SVvalidate (Zheng and Shang, 2024), which specifically re-examines the same long-read alignments (BAM) against the reference genome and the Sniffles2 VCF. This tool leverages long-read sequencing data to verify each predicted SV, thereby reducing false positives and improving confidence in the final SV set (Zheng and Shang, 2024).

### Annotations

#### Repeat annotations

Repetitive sequences in the *T. foetus* TfOz5 genome were identified using a combination of *de novo* and structure-based approaches. RepeatModeler2 v2.0.5 (Flynn et al., 2020) was used to construct a species-specific library of transposable elements (TEs) through *de novo* prediction. This custom TE library was then used by RepeatMasker v4.1.7 (Tarailo-graovac and Chen, 2009) to annotate both known and novel TEs within the genome. The resulting annotations from RepeatModeler2 and RepeatMasker were used to soft mask the draft genome assembly. This masked version of the genome served as the input for gene prediction, thereby reducing the likelihood of erroneous gene models arising from repetitive or low-complexity regions.

#### Gene predictions

Protein-coding genes in the *T. foetus* TfOz5 genome were predicted using the BRAKER3 pipeline (Hoff et al., 2019; Gabriel et al., 2024) after soft-masking repetitive elements with RepeatMasker. Orthologous protein sequences from the OrthoDB database were used as homology-based evidence. No RNA-seq evidence was used for gene prediction. Although a chromosome-scale assembly of KV-1 exists, there is no pre-trained AUGUSTUS model for *T. foetus*, so the gene prediction model required *de novo* training. BRAKER3 v3.0.8 (Lomsadze et al., 2005; Hoff et al., 2016, 2019) integrates GeneMark-ETP v1.02 (Brůna et al., 2024) and AUGUSTUS v3.5.0 (Stanke et al., 2008) in an automated workflow that uses protein evidence to iteratively train models and predict gene structures. Thus, AUGUSTUS training was performed within the BRAKER3 workflow, and no separate post-BRAKER AUGUSTUS training or additional *ab initio* prediction step was performed. The final BRAKER3 gene models were used as the predicted protein-coding gene set for downstream functional annotation. However, since no RNA-seq or long-read transcript evidence was available, exon-intron structures predicted by BRAKER3 were not considered validated spliceosomal introns. In particular, BRAKER3-predicted introns were treated as unsupported ab initio features pending future validation using trichomonad-specific splice-site motif filtering and transcriptomic splice-junction evidence.

#### Functional gene annotations

Functional annotation of the predicted protein-coding genes in the *T. foetus* TfOz5 genome was performed by comparing the predicted protein sequences against multiple functional databases. These included the NCBI non-redundant (NR) protein database, SWISS-PROT (Bairoch and Apweiler, 2000), Gene Ontology (GO) (Ashburner et al., 2000), eggNOG (Huerta-cepas et al., 2019) and KEGG (Kanehisa and Goto, 2000). Sequence similarity searches were conducted using DIAMOND BLASTp (Buchfink et al., 2015) with an E-value threshold of 1.0 × 10^−3^, implemented through the Functional Analysis Module of OmicsBox v3.4.6 (Götz et al., 2008). InterProScan v5.74-105.0 (Jones et al., 2014; Paysan-lafosse et al., 2023) was used to identify conserved protein domains and functional signatures, using the EMBL-EBI version of the InterPro (Paysan-lafosse et al., 2023) database.

#### GO term analysis

Orthologous gene groups shared between *T. foetus* TfOz5 and other trichomonads, as well as closely related species, were identified using OrthoFinder v2.5.5 (Emms and Kelly, 2019). Multiple sequence alignments for each orthogroup were generated with MAFFT v7.526 (Rozewicki et al., 2019) using the L-INS-i algorithm. Gene trees were inferred using IQ-TREE v2.4 (Minh et al., 2020) under the best-fit substitution model selected by ModelFinder, with 1000 ultrafast bootstrap replicates. These gene trees were then used by the STAG algorithm within OrthoFinder to infer the species tree which was subsequently rooted using STRIDE.

Functional differences between *T. foetus* strains TfOz5 and KV-1 and the human protozoan parasite *T. vaginalis* G3 were assessed through GO enrichment analysis of species-specific orthologous genes. For the intra-species comparison (*T. foetus* TfOz5 vs KV-1), 2 test sets were defined: (i) genes unique to TfOz5 and (ii) genes unique to KV-1 as identified by OrthoFinder clustering. For the inter-species comparison (*T. foetus* TfOz5 vs *T. vaginalis* G3), conserved and divergent sets were derived from orthogroups containing genes unique to each species after excluding shared orthologues. GO annotations for protein-coding genes were assigned using InterProScan v5.74-105.0 (Jones et al., 2014; Paysan-lafosse et al., 2023) integrated within OmicsBox v3.4.6 (Götz et al., 2008), incorporating multiple databases such as Pfam v37.0 (Mistry et al., 2021), SMART (Letunic et al., 2021) and PROSITE (Sigrist et al., 2013) to predict functional domains and associated GO terms.

Enrichment analysis was conducted in OmicsBox v3.4.6 (Götz et al., 2008) using Fisher’s exact test comparing the frequency of GO terms in each species-specific set against a background consisting of all annotated protein-coding genes in the TfOz5 genome. Multiple testing correction was applied using the Benjamini–Hochberg False Discovery Rate (FDR) method and GO terms with an adjusted *p*-value < 0.05 were considered significantly enriched. Significant terms were categorized into the 3 main GO domains: Biological Process (BP), Molecular Function (MF) and Cellular Component (CC).

## Results

### Tritrichomonas foetus *TfOz5 sequencing and k-mer-based approximation of genome size*

Oxford Nanopore, Illumina and Pore-C sequencing produced high-quality datasets for genome assembly (Table 1). GenomeScope 2.0 estimated a haploid genome size of 146.3 Mb with 42% unique content, indicating a high proportion of repetitive elements. The final chromosome-scale TfOz5 assembly was 137.7 Mb, approximately 8.6 Mb shorter than the GenomeScope estimate and ∼11 Mb shorter than the published KV-1 assembly. The difference between the GenomeScope estimate, the TfOz5 assembly and the published KV-1 assembly indicates that assembly-size variation remains unresolved in the current analysis. Since TfOz5 and KV-1 differ in sequencing strategy, scaffolding approach, repeat resolution and assembly processing, the smaller TfOz5 assembly cannot be attributed solely to biological genome-size differences or repeat collapse without additional whole-genome alignment and repeat-focused comparison. Repeat annotation showed that 43.6% of the TfOz5 assembly consists of repetitive elements, supporting the likelihood that repetitive sequence contributes to uncertainty in assembly size. However, repeat content alone does not identify which regions account for the size difference relative to GenomeScope or KV-1. The final assembly is therefore presented as a high-quality chromosome-scale representation of TfOz5, while recognizing that some repeat-rich regions may remain collapsed, unresolved or differentially represented relative to KV-1 and the k-mer-based genome-size estimate. The k-mer profile showed a dominant coverage peak at ∼20×, low heterozygosity (0.467%) and a duplication rate of 0.566%, confirming the quality of the sequencing data (Figure S1a). Summary statistics for the Pore-C run which provided chromatin contact information for scaffolding are presented in Table S2.

**Table 1.** Statistics of raw and quality filtered sequencing data used for *T. foetus* TfOz5 assembly Table 1 long description.

Sequencing libraries (platform)	Number of reads	Number of bases	Average read length	Q20 (%)
**WGS long reads (ONT PromethION)**	822,732	7,770,207,935	9444	88.43
**WGS long reads + Quality filtering^a^**	209,038	7,336,486,177	35,097	89.65
**WGS short reads (Illumina NovaSeq)**	55,490,372	16,647,111,600	150	99.17
**WGS short reads + Quality filtering^a^**	55,010,774	16,393,210,652	149	99.38
**Pore-C (ONT PromethION)**	771,212	438,090,484	568	88.72
**Pore-C + Quality filtering^a^**	757,633	400,801,765	529	90.12

a Quality filtering of the nanopore reads employed Porechop (default settings) and NanoFilt (20 kilobase pairs and *Q* score < 12) while quality filtering for the Illumina genomic DNA library employed fastp program (default parameters).

### Chromosome-scale genome assembly of Tritrichomonas foetus TfOz5

**Figure 1.**
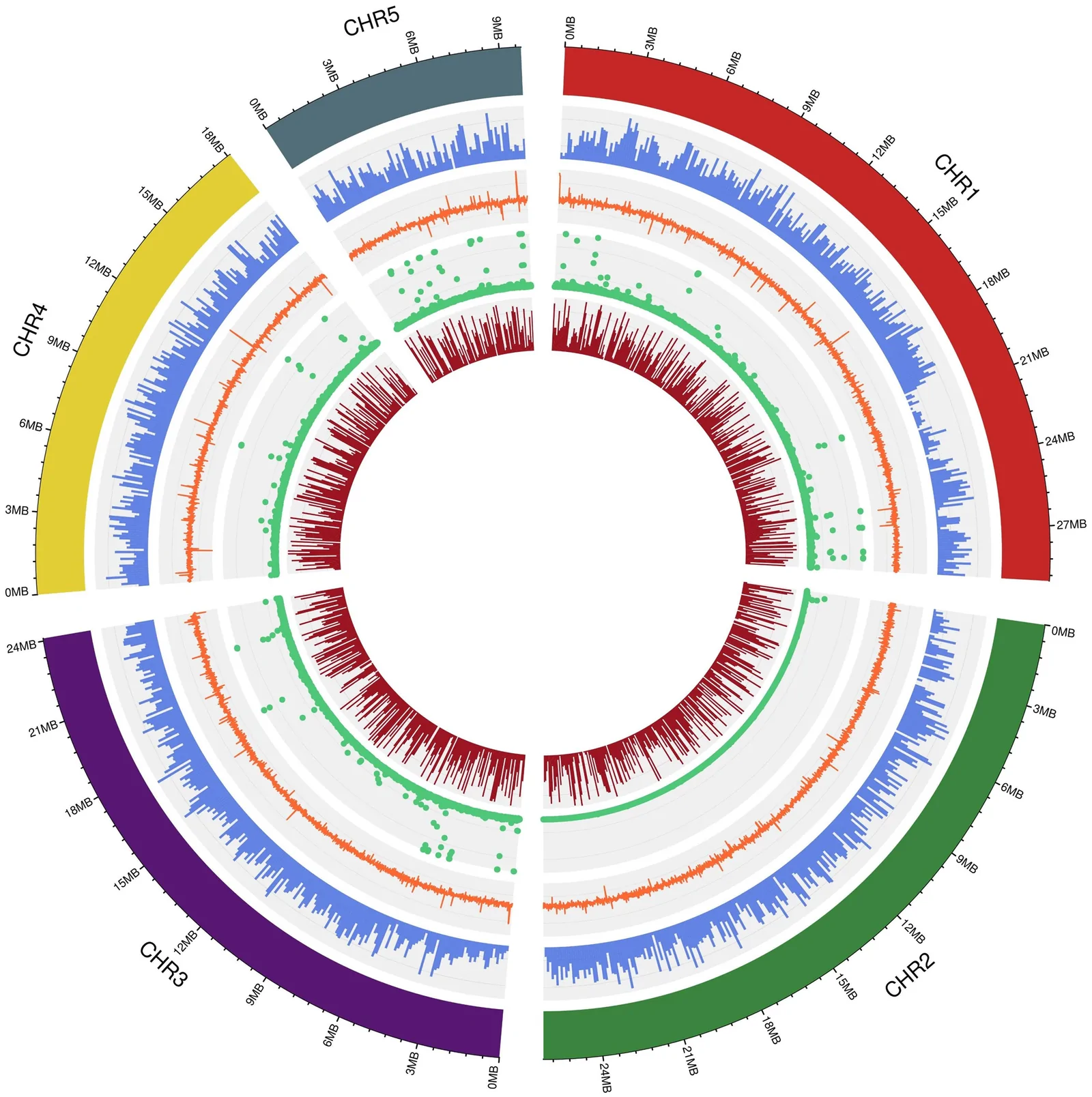
Circos plot of the *Tritrichomonas foetus* TfOz5 genome illustrating multi-layered genomic features across the 5 chromosomes CHR1 to CHR5. The outermost ring is the chromosomes in the TfOz5 genome. The second ring displays gene density as blue bars, highlighting regions of varying gene content. The third layer presents GC skewness as an orange line, indicative of potential replication-related patterns. Green dots in the fourth layer represent the N-ratio, marking regions with ambiguous or low-confidence base calls. In the fifth layer, red bars denote sequencing coverage, identifying well-covered regions and potential gaps in sequencing depth. Table 1 long description.

The *de novo* genome assembly was based on long-read assembly followed by polishing with short reads to ensure the contiguity and accuracy of the genome. Pore-C contact mapping was used to scaffold the polished assembly to chromosome scale (Figure S1b). The final assembly comprised 58 scaffolds in total, including 5 chromosome-scale scaffolds corresponding to CHR1–CHR5, with the remaining smaller scaffolds retained as unplaced/unanchored sequences. The assembly had an N50 of 24 Mb and a total genome size of 137.7 Mb. Telomeric repeats were not confirmed at all chromosome ends; therefore, the assembly is described as chromosome-scale rather than telomere-to-telomere complete. This represents a greater than 275-fold improvement in scaffold contiguity compared to the previously published reference genome *T. foetus* K strain assembly (1479 scaffolds, N50 = 86.5 kb), and a notable advance over the *T. foetus* KV-1 assembly (578 scaffolds, N50 = 22 Mb). The spatial organization of genomic features in the *T. foetus* TfOz5 genome is shown in Figure 1. Across the 5 chromosomes, the circos tracks reveal several clear patterns (Figure 1). Most notably, Chromosome 2 (CHR2) displays a consistently low N-ratio in the green track indicating fewer ambiguous base calls and consequently higher consensus confidence than the other chromosomes. This signal coincides with uniformly high read coverage (red bars) suggesting that CHR2 was resolved with fewer assembly uncertainties.

### Comparative genomics with T. foetus isolates and other Parabasalia genomes

To contextualize the structural and genomic features of the *T. foetus* TfOz5 assembly, we compared it with 3 publicly available *T. foetus* genomes: KV-1, K strain and the Belfast isolate selected for their relevance and diversity in assembly quality and sequencing strategies (Table 2). Among these, TfOz5 stands out as the most contiguous and structurally resolved assembly, reaching chromosome-level organization. It also shows the highest BUSCO completeness, underscoring its value as a reference for downstream analyses. In contrast, the other assemblies vary in contiguity and completeness, reflecting differences in sequencing technologies and assembly approaches. Overall, this comparison highlights the technological advancements that underpin the improved genome contiguity of TfOz5. Key metrics such as scaffold N50, contig count and completeness are summarized in Table 2 which also shows that GC content remains consistent across isolates and related *Trichomonad* species.

**Table 2.** Comparative genomics for *Tritrichomonas foetus* TfOz5 and other *Parabasalia* genomes Table 2 long description.

Metric	*T. foetus* TfOz5	*T. foetus* KV-1	*T. vaginalis* G3	*T. foetus* K	*H. meleagridis* 2922-C6/04-10x	*T. foetus* Belfast	*T. tenax* Hs-4:NIH	*T. gallinae* TGAL	*T. musculus* EAF2021
Assembly level	Chromo-some	Chromo-some	Chromo-some	Scaffold	Scaffold	Contig	Contig	Contig	Contig
GenBank accession	JBVWGG000000000	GCA_041260205.1	GCA_026262505.1	GCA_001839685.2	GCA_020186115.1	GCA_905133005.1	GCA_023091735.1	GCA_047301465.1	GCA_039105265.1
Sequencing technology	Oxford Nanopore; Illumina; Pore-C	Oxford Nanopore; Illumina; Hi-C	PacBio; Illumina; Hi-C	Illumina	Oxford Nanopore; Illumina	PacBio	Illumina	Illumina	PacBio; Oxford Nanopore
Genome size	137.7 Mb	148.7 Mb	181 Mb	67.6 Mb	43.4 Mb	147 Mb	63.4 Mb	55.7 Mb	193.5 Mb
Chromosome	5	5	6	N/A	N/A	N/A	N/A	N/A	N/A
Scaffold No.	58	578	218	1479	187	N/A	N/A	N/A	N/A
Scaffold N50	24 Mb	22 Mb	27.7 Mb	86.5 kb	673.5 kb	N/A	N/A	N/A	N/A
Scaffold L50	3	3	3	243	16	N/A	N/A	N/A	N/A
Contigs	194	693	858	3298	188	2776	4904	8276	756
Contig N50	2 Mb	2 Mb	541.2 kb	34.8 kb	673.5 kb	82.2 kb	38.4 kb	28.1 kb	3.6 Mb
Gaps (%)	0.003	0.01	0.02	3.38	N/A	N/A	N/A	N/A	N/A
GC (%)	31.02	30.75	32.5	31	28.5	31	33.9	33.5	29.5
Genome coverage	77.8x	76x	180x	73x	200x	60x	50x	100x	26x
Genome completeness (%)	52.9	52.6	51.8	50.6	52.1	42.7	49.8	48.7	53.4
Reference	This study	Abdel-glil et al., 2024	Sullivan et al., 2025	Benchimol et al., 2017	Palmieri et al., 2021	Senior, 2021	Yang et al., 2022	Sullivan et al., 2025	Alves-Ferreira et al., 2025

N/A: Data are not applicable, or information is not available.

Although TfOz5 performs favourably in comparative BUSCO completeness relative to other available parabasalid/trichomonad assemblies (Table 2), it is important to note that BUSCO completeness values across *Parabasalia* remain modest in absolute terms. This pattern likely reflects a combination of (i) reference-set limitations in eukaryote-wide BUSCO datasets for divergent parabasalids and (ii) annotation challenges for small or highly divergent genes in repeat-rich genomes, rather than indicating poor chromosome-scale assembly structure.

### Repeat content

Repeat annotation of the *T. foetus* TfOz5 genome revealed that repetitive elements account for over 43% of the assembly (Table 3). DNA transposons were the most abundant repeat class followed by a large proportion of unclassified interspersed repeats. Retroelements, simple sequence repeats and low-complexity regions occurred at lower frequencies while no satellite DNA or rolling-circle transposons were detected. Compared to the KV-1 genome, TfOz5 contains a lower overall repeat content (43.6% vs. 50.2%), driven primarily by a reduced proportion of DNA transposons (24.6% vs. 32.5%) and retroelements (0.5% vs. 2.9%). Notably, TfOz5 harbours Penelope elements which were not reported in KV-1 and includes small RNA-associated repeats absent from KV-1. However, these differences should be interpreted cautiously since variation in repeat content between assemblies may also reflect technical factors, including differences in assembly completeness, repeat resolution, repeat-library construction and annotation parameters. Therefore, the apparent differences in repeat composition between TfOz5 and KV-1 are reported as putative differences and require confirmation using harmonized repeat annotation approaches and additional *T. foetus* genomes.

**Table 3.** Comparison of repeat content between *T. foetus* TfOz5 and KV-1 Table 3 long description.

	Number of elements		Length (bp)		Proportion in genome (%)	
Repeat element	TfOz5	KV-1^a^	TfOz5	KV-1^a^	TfOz5	KV-1^a^
Retroelements	564	625	691,637	4,238,700	0.52	2.85
SINE	159	118	71,806	19,701	0.05	0.76
Penelope	92	NR	25,866	NR	0.02	NR
LINE	181	101	419,782	330,525	0.31	0.22
LTR elements	224	1714	200,049	777,456	0.15	0.52
DNA transposons	12,373	20,695	33,002,701	48,379,606	24.64	32.53
Rolling circles	0	0	0	0	0	0
Unclassified	33,010	33,697	24,715,458	25,079,482	18.45	16.86
**Total interspersed repeats**			58,435,662	74,586,770	43.62	50.15
Small RNA	159	0	71,806	0	0.05	0
**Tandem repeats**						
Simple repeats	6328	23,238	320,262	1,214,291	0.24	0.82
Low complexity	1721	5875	133,120	354,663	0.10	0.24

a *Tritrichomonas foetus* KV-1 repeat content published by Abdel-Glil et al., (2024).

NR: Not reported.

### Functional and structural genome annotation

Gene prediction identified a total of 32 104 protein-coding genes in the *T. foetus* TfOz5 genome. The average gene length was 1787 bp, with gene sizes ranging from 201 bp to 29 748 bp. BRAKER3 initially predicted 24 395 monoexonic gene models and 7709 multi-exonic gene models containing 7715 putative introns. However, since the BRAKER3 annotation was generated using protein evidence only and did not include RNA-seq, Iso-Seq or trichomonad-specific splice-motif validation, these exon–intron structures were not considered validated spliceosomal introns. Given that trichomonad genomes are generally intron poor (Callejas-hernández et al., 2025), the BRAKER3-derived intron count is likely inflated and should be interpreted as unsupported gene-model structure rather than confirmed intron annotation. We therefore report the TfOz5 annotation primarily as a predicted protein-coding gene set, while treating the predicted multi-exonic models and putative introns as provisional features requiring future validation with transcriptomic splice-junction evidence and trichomonad-specific intron motif filtering.

**Figure 2.**
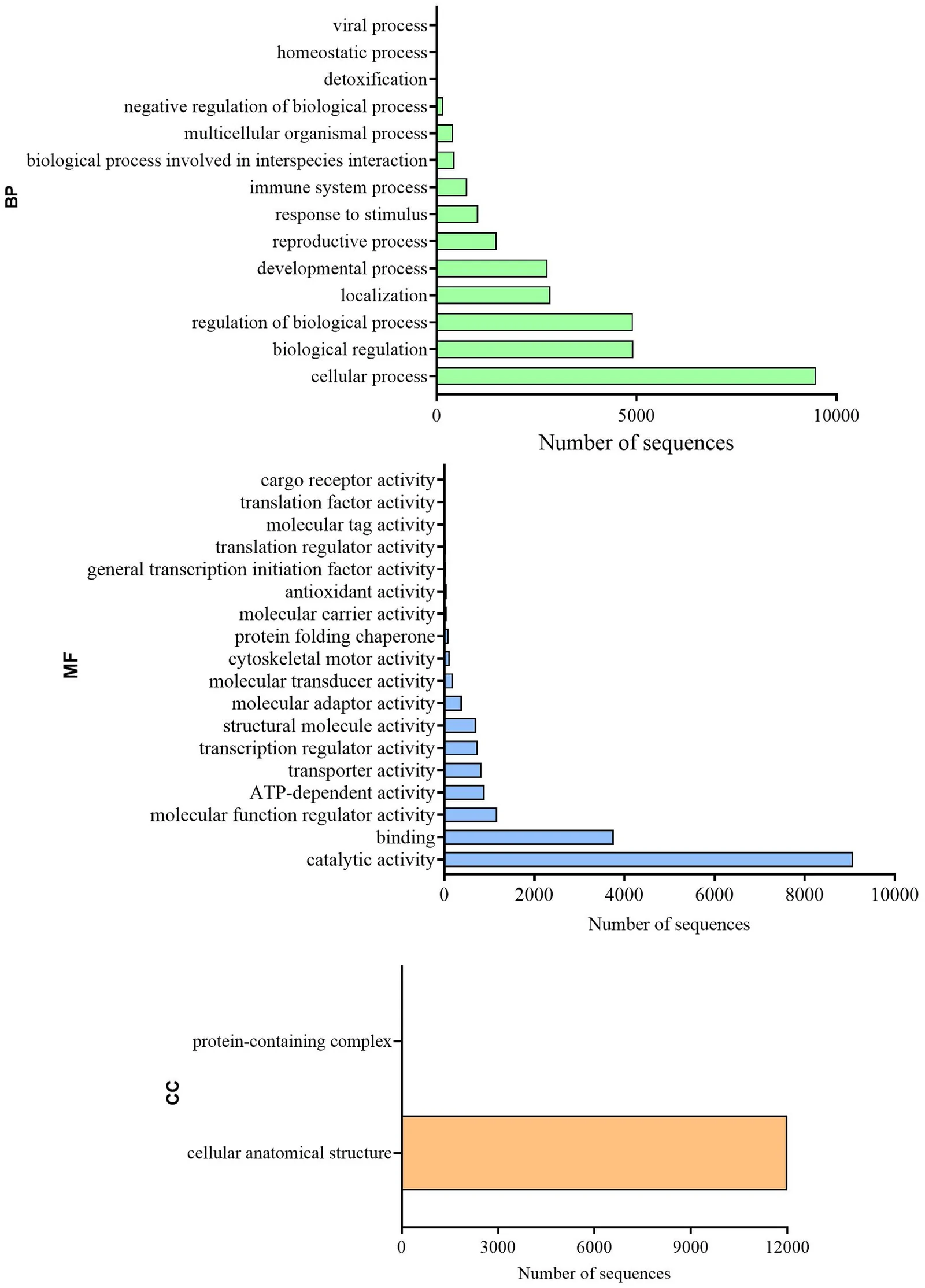
Bar plots depicting the Gene Ontology (GO) classification of functionally annotated genes in the *Tritrichomonas foetus* TfOz5 genome. The distributions of GO terms are presented within the 3 main GO domains including ‘biological process (BP)’ (green), ‘molecular function (MF)’ (blue) and ‘cellular component (CC)’ (orange). Figure 2 long description.

Of the 32 104 predicted protein sequences, 30 373 had BLAST hits in the NR database, 22 712 in the RefSeq database and 10 309 in the SWISS-PROT database. InterProScan analysis identified conserved domains and functional signatures in 31 990 predicted protein sequences, of which 19 295 (60.1%) were associated with Gene Ontology terms and 9313 (48.3%) with Enzyme Code annotations (Table S3). The GO classification of the functionally annotated genes within 3 main GO domains showed that 29 284 of the genes belong to ‘biological process,’ 18 207 to ‘molecular function,’ and 12 025 to ‘cellular component’ (Figure 2, Table S4). The most prominent GO terms at level 2 for biological process were ‘cellular process’ (*n* = 9485) and ‘biological regulation’ (*n* = 4915). Similarly, for the cellular component domain, the main GO classes identified were ‘cellular anatomical structure’ (*n* = 11 998) and ‘protein-containing complex’ (*n* = 27). For molecular function GO terms at level 2, ‘catalytic binding’ (*n* = 9070) and ‘binding’ (*n* = 3760) were the primary classes involved. A linear genome feature map (Figure S2, Table S4) illustrates the spatial distribution of genes, repeats and structural elements across the 5 chromosomes.

### Structural variant landscape in the TfOz5 genome

A comprehensive analysis of the *T. foetus* TfOz5 genome using KV-1 as the reference identified 160 SVs across its 5 chromosomes, spanning 107.1 Mb. These included 65 deletions (40.6%), 36 insertions (22.5%), 26 duplications (16.3%), 21 inversions (13.1%) and 12 translocations (7.5%). SVs were unevenly distributed, with chromosome 1 containing the most (43; 26.9%) followed by chromosome 5 (37; 23.1%), chromosome 2 (28, 17.5%), chromosome 4 (27, 16.9%) and chromosome 3 (25, 15.6%). Deletions were the most frequent SV type on all chromosomes, while insertions and duplications were also common. Inversions and translocations were less frequent but present on every chromosome, indicating that all major SV classes have contributed to differences observed between the *T. foetus* TfOz5 and KV-1 strains (Table S5-S6).

The size distribution of SVs (Table S7) revealed substantial diversity among variant types. Deletions exhibited the broadest size range, spanning from spanning 0.5–12 kb, with a median size of 2.3 kb and an interquartile range (IQR) of 1.1–4.8 kb. Insertions ranged from 0.4 to 8 kb (median 1.7 kb, IQR 0.9–3.2 kb), while duplications varied from 0.6 to 10 kb (median 2.1 kb, IQR 1.0–4.0 kb). Inversions were generally smaller, ranging from 0.7 to 6 kb (median 1.9 kb, IQR 1.0–2.8 kb), and translocations were the most narrowly distributed, spanning 0.8 to 5 kb (median 1.5 kb, IQR 1.0–2.0 kb).

The impact of the SVs in the *T. foetus* TfOz5 genome revealed that 142 of the 160 SVs overlapped with annotated genes affecting 1026 gene models across all 5 chromosomes. These effects consisted of complete gene loss in 312 genes (30.4%), partial disruptions in 598 genes (58.3%) and minor overlaps in 116 genes (11.3%). As larger chromosomes typically contain more genes, higher raw counts are expected for chromosome 1 and chromosome 2 (278 and 221 SV-impacted genes, respectively). After normalizing by chromosome size, the rate of SV-impacted genes per 1000 annotated genes is as follows: chromosome 1 (116; 11.6%), chromosome 2 (62; 6.2%), chromosome 5 (59; 5.9%), chromosome 4 (57; 5.7%) and chromosome 3 (50; 5.0%). These results indicate broadly comparable rates (5–6%) across chromosomes 2 to 5 and a genuinely higher impact density on chromosome 1. Functional annotation indicated enrichment for genes encoding adhesins (AP65 family), cysteine proteases (CP) and metabolic enzymes among disrupted genes. These disruptions were caused by deletions resulting in complete gene loss. These affected functions implicate roles in host interaction, virulence and immune evasion. Overall, the SV landscape provides genomic evidence that structural rearrangements may influence the repertoire of genes implicated in host interaction; however, whether these SVs alter expression, localization or phenotypic virulence requires targeted experimental testing (RT-qPCR/RNA-seq to test transcript loss or altered isoforms in SV-positive versus SV-negative isolates; surface/secretome proteomics to confirm protein expression and accessibility; and functional assays such as epithelial adherence inhibition and protease activity assays). Where causal inference is required, transfection-based approaches (including stable transfection/heterologous expression demonstrated in *T. foetus*) can be used for complementation or overexpression, with gene knockdown or CRISPR/Cas systems established in related trichomonads providing a template for future adaptation. SVs appear to contribute substantially to gene content diversity in *T. foetus* TfOz5 although the functional consequences of specific SVs remain to be validated experimentally. Detailed results are presented in Table S8.

### Comparative genomic analysis of Parabasalia using Orthofinder

A comprehensive comparative genomic analysis was conducted using OrthoFinder across 9 *Parabasalia* species: *Histomonas meleagridis, Pentatrichomonas hominis, Trichomonas gallinae, Tritrichomonas musculus, Trichomonas tenax, Trichomonas vaginalis G3* (Sullivan et al., 2025)*, Tritrichomonas foetus K, T. foetus KV-1* and *T. foetus TfOz5*. This analysis enabled the characterization of evolutionary relationships, the extent of gene family conservation and the identification of species-specific genes.

**Figure 3.**
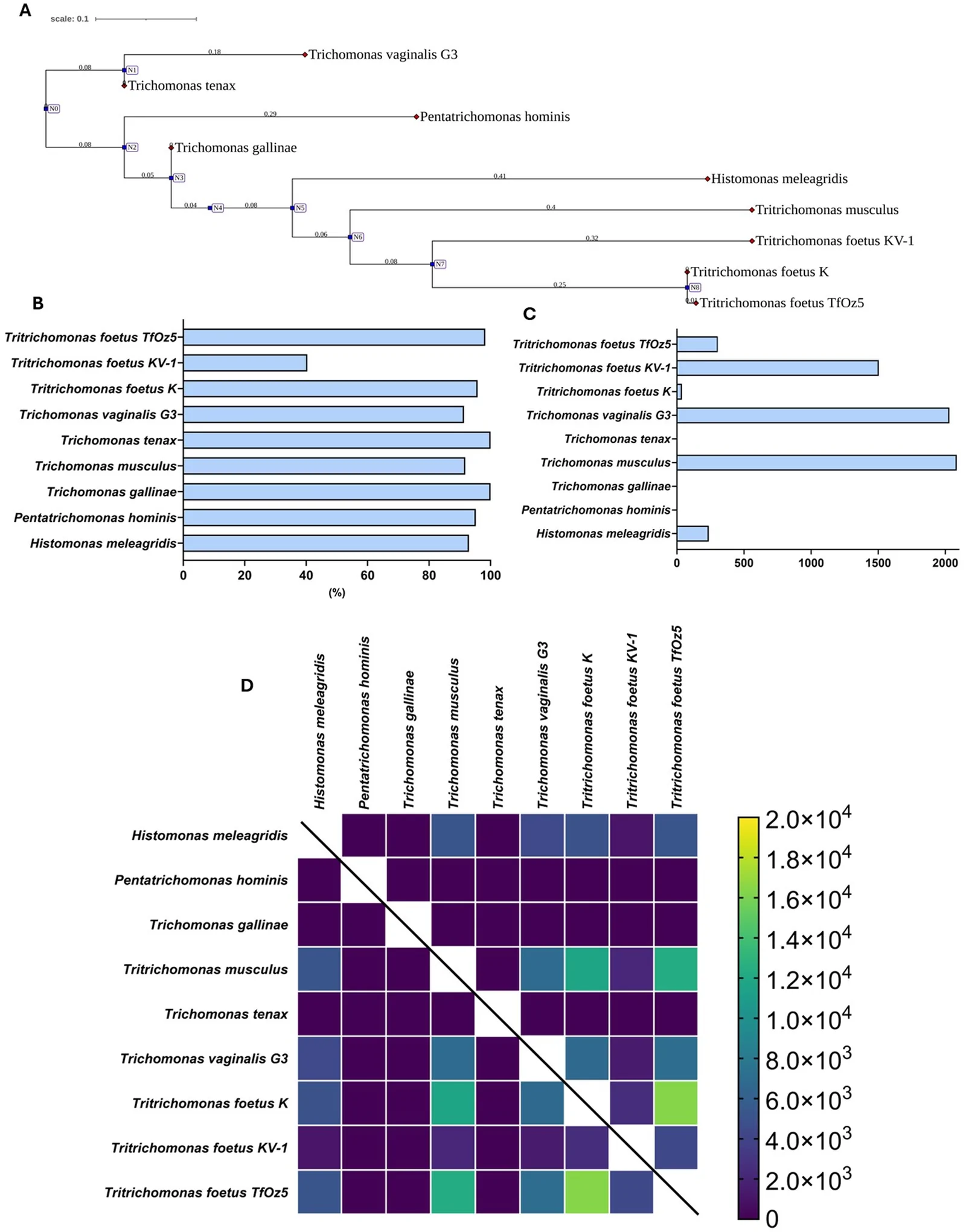
Summary of OrthoFinder analysis in set of *Parabasalia* species: *Histomonas meleagridis, Pentatrichomonas hominis, Trichomonas gallinae, Tritrichomonas musculus, Trichomonas tenax, Trichomonas vaginalis* G3, *Tritrichomonas foetus* K, *Tritrichomonas foetus* KV-1 and *Tritrichomonas foetus* TfOz5. (A) The phylogenetic tree inferred by STAG and rooted by STRIDE. (B) Percentage of genes from each species assigned to orthogroup. (C) Number of species-specific orthogroups. (D) Number of orthogroups containing species. Figure 3 long description.

The phylogenetic tree, inferred using the STAG algorithm and rooted with STRIDE, revealed well-supported evolutionary relationships among the taxa (Figure 3a). The 3 *T. foetus* strains (K, KV-1 and TfOz5) formed a monophyletic cluster, indicative of high genomic similarity, while *Tritrichomonas musculus* was resolved as a distinct lineage, highlighting its divergence within the *Tritrichomonas* clade. To ensure robust gene tree inference, multiple sequence alignments for each orthogroup were generated using MAFFT L-INS-i and analysed with IQ-TREE under the best-fit substitution model selected by ModelFinder, with 1000 ultrafast bootstrap replicates. These high-quality gene trees were then used by STAG to construct the species tree, improving accuracy compared to default FastTree-based workflows.

Analysis of gene assignment to orthogroups demonstrated a high degree of conservation across most species (Figure 3b). Complete orthogroup assignment was observed in *T. gallinae* and *T. tenax*, with 100% of genes assigned. Similarly, high proportions were recorded for *T. musculus* (91.8%), *H. meleagridis* (93.0%), *P. hominis* (95.2%), *T. vaginalis* G3 (91.4%) and *T. foetus* K (95.8%). *Tritrichomonas foetus* TfOz5 also exhibited a high assignment rate (98.3%), reinforcing the presence of a conserved genomic core. In contrast, *T. foetus* KV-1 displayed a markedly lower orthogroup assignment rate (40.4%), despite having ∼10 000 more predicted genes than TfOz5.

The identification of species-specific orthogroups (gene families unique to individual species) provided further insight into lineage-specific genes (Figure 3c). *Tritrichomonas musculus* exhibited the highest number of species-specific orthogroups (2085), indicating extensive gene family diversification. *Trichomonas vaginalis* G3 also harboured a substantial number (2029), followed by *T. foetus* KV-1 (1107) and *T. foetus* TfOz5 (305). In contrast, *P. hominis* and *T. tenax* lacked species-specific orthogroups, and *T. gallinae* possessed only 1, suggesting a more conserved gene repertoire in these lineages.

Analysis of orthogroup distribution revealed distinct patterns of gene family conservation and divergence among trichomonad species (Figure 3d). *Tritrichomonas foetus* TfOz5 shared the highest number of orthogroups (16 366) with *T. foetus* K, indicating strong genomic similarity. *Trichomonas vaginalis* G3 and *T. foetus* TfOz5 shared 7013 orthogroups, while *T. foetus* KV-1 shared 4299 orthogroups with TfOz5, suggesting moderate divergence among *T. foetus* strains.

### Conserved and divergent genes between Tritrichomonas foetus TfOz5 and KV-1, and between TfOz5 and Trichomonas vaginalis G3

**Figure 4.**
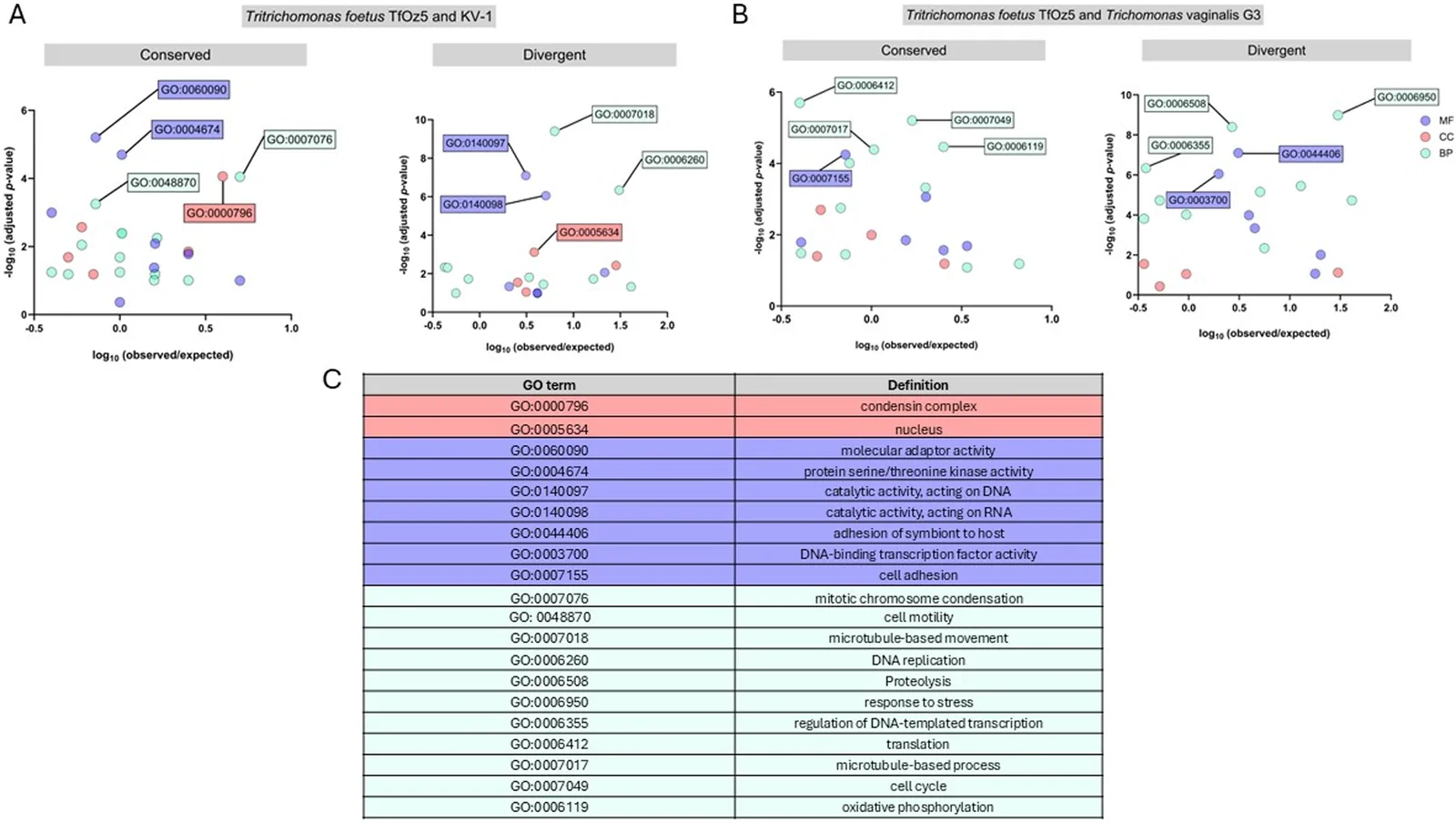
Gene Ontology (GO) enrichment analysis of conserved and divergent genes between *Tritrichomonas foetus* strains (TfOz5, KV-1) and *Trichomonas vaginalis* G3. (A) Scatter plots showing the log₁₀ (observed/expected) ratio versus −log₁₀ (adjusted *p*-value) for enriched GO terms in conserved (left) and divergent (right) gene sets. (B) Scatter plots for conserved (left) and divergent (right) gene sets between *T. foetus* TfOz5 and *T. vaginalis* G3. Each point represents a GO term, color-coded by category: Biological Process (BP), Molecular Function (MF) or Cellular Component (CC). Significant GO terms (adjusted *p*-value < 0.05) are highlighted. (C) Table summarizing the definitions and categories of the highlighted GO terms. Figure 4 long description.

GO enrichment analysis revealed functional differences between conserved and divergent gene sets in both intra-species (*T. foetus* TfOz5 and KV-1) (Figure 4a) and inter-species (*T. foetus* TfOz5 and *T. vaginalis* G3) comparisons (Figure 4b). In the comparison of *T. foetus* TfOz5 and KV-1, conserved genes were enriched for condensin complex (GO:0000796), cell motility (GO:0048870), mitotic chromosome condensation (GO:0007076), protein serine/threonine kinase activity (GO:00004674) and molecular adaptor activity (GO:0060090), suggesting roles in chromosome organization, cytoskeletal dynamics and signal integration that may contribute to parasite survival. Divergent genes were enriched for nucleus (GO:0005634), catalytic activity acting on RNA (GO:0140098), catalytic activity acting on DNA (GO:0140097) and DNA replication (GO:0006260) suggesting functional divergence in transcriptional and replication processes.

For the comparison between *T. foetus* TfOz5 and *T. vaginalis* G3 (Figure 4b), conserved genes were enriched for translation (GO:0006412), cell cycle (GO:0007049), oxidative phosphorylation (GO:0006119) and cell adhesion (GO:0007155), suggesting conservation of core metabolic and cytoskeletal organization (microtubule-based processes/microtubule organization). Divergent genes, on the other hand, were enriched for proteolysis (GO:0006508), response to stress (GO:0006950), regulation of DNA-templated transcription (GO:0006355) and adhesion of symbiont to host (GO:0044406), suggesting that these divergent functions may contribute to species-specific interactions with its respective host environment and adaptation to the specific conditions of its ecological niche (bovine reproductive tract versus human urogenital tract). All enriched terms were significant after FDR correction (adjusted *p*-value < 0.05).

## Discussion

This study presents a high-quality, chromosome-scale genome assembly of *T. foetus* TfOz5, constructed using Pore-C and Oxford Nanopore long-read sequencing. The resulting assembly spans 5 chromosomes with minimal gaps, offering a structurally resolved and highly contiguous genome. This development significantly enhanced the genomic resources available for *T. foetus*, thereby facilitating future comparative, functional and evolutionary investigations of this economically significant bovine parasite. While the KV-1 assembly provides a valuable genomic resource, the TfOz5 genome offers enhanced contiguity and structural resolution through Pore-C and long-read sequencing. Importantly, TfOz5 represents a geographically distinct *T. foetus* Australian strain. This assembly not only complements KV-1 but also expands the genomic toolkit for comparative, functional and epidemiological studies of *T. foetus*.

Although TfOz5 was derived from a single field isolate stock recovered from a single bull and was not generated by pooling multiple isolates, it was not cloned from a single parasite cell. This distinction is relevant since available *T. foetus* genome assemblies, including the K, KV-1 and TfOz5 strains, appear to have been generated from cultured isolates or strains, and the published descriptions do not always establish whether those cultures originated from single-cell cloned parasite populations. Therefore, these assemblies are best interpreted as cultured isolate genomes rather than definitive representations of all parasite diversity originally present in the host. For TfOz5, the low k-mer-based heterozygosity and duplication estimates are consistent with a single dominant genotype in the sequenced material, with no obvious signal of a highly mixed parasite population. However, the extent of within-host genetic diversity of *T. foetus* in naturally infected bulls remains poorly understood. If mixed infections occur, *in vitro* culture and repeated passage may preferentially select faster-growing genotypes and reduce detectable diversity, meaning that culture-derived assemblies may represent the dominant lineage rather than the complete parasite population present in the original host. Our preliminary unpublished comparison with another geographically distinct Australian *T. foetus* isolate showed high similarity to TfOz5 (∼99.2%), which may be consistent with limited diversity among the Australian isolates sampled to date; however, this observation requires confirmation and should not be overinterpreted. Future direct-from-sample sequencing, population genomic analyses across additional Australian and international isolates, and, where feasible, single-cell approaches will be required to determine whether mixed *T. foetus* infections occur, quantify within-host diversity and assess broader population structure.

The *T. foetus* TfOz5 genome demonstrates higher contiguity and genome completeness compared to previously published assemblies, including the chromosome-level *T. foetus* KV-1 genome and the draft K strain making it the most contiguous *T. foetus* assembly to date. These comparative results highlight the improved quality and completeness of the *T. foetus* TfOz5 assembly, which is particularly advantageous for downstream structural and comparative genomic analyses. The enhanced contiguity and resolution of the *T. foetus* TfOz5 genome can be attributed to the integration of Pore-C. Pore-C provides richer 3D chromatin information than Hi-C by sequencing multi-way contacts on long fragments. It reduces computational assumptions and preserves native methylation. This likely explains the improved contiguity of TfOz5 (58 scaffolds, N50 24 Mb) compared to KV-1 (578 scaffolds, N50 22 Mb) even though both are chromosome-level assemblies. Unlike traditional Hi-C, Pore-C captures multi-way chromatin interactions, providing more comprehensive spatial information that facilitates accurate scaffolding, especially in regions with complex structural features or high repeat content (Deshpande et al., 2022; Nguyen Loan et al., 2023; Ong et al., 2024). Although Pore-C has not previously been applied to *T. foetus*, its efficacy has been demonstrated in various eukaryotic systems, including humans cell lines (Deshpande et al., 2022; Zhong et al., 2023) and agricultural species such as *B. ovis* and *Bos indicus* (Nguyen Loan et al., 2023; Ong et al., 2024). These precedents highlight the versatility of Pore-C and its potential to improve genome assembly and annotation across diverse taxa.

BUSCO values were interpreted cautiously since currently available BUSCO datasets are not optimized for divergent parabasalids/trichomonads. Therefore, BUSCO was retained only as a broad comparative metric and was not used as the primary measure of assembly or annotation completeness. Repetitive sequences constitute 43.62% of the *T. foetus* TfOz5 genome, slightly lower than the 48.93% reported in *T. foetus* KV-1 (Abdel-glil et al., 2024). This high repeat content is consistent with patterns observed in parasitic protozoa where repetitive DNA play central roles in genome plasticity, adaptation and evolution (Homan et al., 2000; Wickstead et al., 2003; Abdel-glil et al., 2024; Dickson et al., 2025). In both *T. foetus* TfOz5 and KV-1, DNA transposons dominate the repetitive landscape (Abdel-glil et al., 2024), suggesting that transposable elements have historically shaped genome architecture through insertional mutagenesis and recombination (Mizuuchi, 1992; Mizuuchi et al., 1992; Kidwell and Lisch, 2001; Kazazian, 2004). DNA transposons can insert into different genomic locations, potentially disrupting genes or regulatory sequences (insertional mutagenesis) (Mizuuchi, 1992; Kidwell and Lisch, 2001; Kazazian, 2004). They can also mediate recombination events, leading to larger-scale structural changes like deletions, inversions and translocations (Kazazian, 2004).

Comparative analysis with other parasitic protozoa reinforces the evolutionary significance of repetitive DNA. For instance, original draft genome of the genome of *T. vaginalis* is exceptionally rich in repetitive elements estimated at ∼65% (Carlton et al., 2007). These include DNA transposons, LINE-like retrotransposons and other mobile elements implicated in gene family expansion and genome rearrangement (Carlton et al., 2007). Other protozoan parasites, such as *Entamoeba histolytica* (20%) (Lorenzi et al., 2008), *Trypanosoma brucei* (30%) (Van Leeuwen et al., 1996) and *Giardia lamblia* (40%) (Sun et al., 2010), also exhibit substantial repeat content, albeit with species-specific differences. These comparisons suggest that while the composition and abundance of repeats vary, they continue to have a role as key drivers of genome evolution and adaptability.

In addition to repeat content, comparison of the SV landscape between *T. foetus* TfOz5 and the KV-1 genome reveals a complex and heterogeneous pattern of genomic rearrangements. We identified 160 SVs across the 5 chromosomes of TfOz5, including deletions, insertions, duplications, inversions and translocations. SVs were unevenly distributed across TfOz5 chromosomes (CHR1: 43; CHR5: 37; CHR2: 28; CHR4: 27 and CHR3: 25), and all SV classes (deletions, insertions, duplications, inversions and translocations) were represented on every chromosome, with deletions predominating on each. Since CHR1 and CHR2 are the largest chromosomes, raw counts partly track chromosome size; we therefore normalized by length to enable direct comparison across. This framing allows us to distinguish true SV hotspots from size effects and to interpret per-chromosome differences rather than raw counts alone. Deletions were the most prevalent (40.6%), consistent with observations in other protozoan parasites such as *T. brucei* and *Plasmodium falciparum* where deletions are often linked to gene loss or modulation of gene expression (Maltha et al., 2012; Kumar et al., 2013; Kirkman and Deitsch, 2014; Florini et al., 2022). These SVs which differ from those in KV-1 may disrupt gene regulation or drive chromosomal rearrangements that influence genome stability. Interpretation of SV impacts is necessarily limited by the absence of direct functional assays in this study. Although many SVs overlap genes annotated as adhesins, cysteine proteases and other candidate virulence-associated factors, these genomic overlaps alone do not demonstrate that SVs change expression, protein localization or host-interaction phenotypes. Accordingly, the results are best interpreted as patterns consistent with SV contributing to diversification of host-interaction repertoires, and they motivate targeted functional follow-up rather than supporting definitive causal claims. Although several structural variants overlapped genes annotated with potential host-interaction functions, these associations remain hypothesis-generating and require experimental validation.

To contextualize these findings within a broader evolutionary framework, a comparative genomic analysis of 9 *Parabasalia* species was conducted using OrthoFinder. This analysis provided an orthogroup-informed comparative genomics context, summarizing broad clustering among the sampled genomes, gene family conservation, lineage-specific genes and patterns of orthogroup sharing. Since OrthoFinder infers a species tree from genome-wide orthogroups (e.g., via STAG with rooting via STRIDE) and as taxon sampling was limited to 9 genomes, these results should be interpreted as an approximation of relationships among the sampled taxa rather than a definitive reconstruction of deep *Parabasalia* phylogeny; broader phylogenomic/taxonomic syntheses are addressed in phylum-wide studies (Boscaro et al., 2024). Phylogenetic reconstruction grouped the 3 *T. foetus* strains: K, KV-1 and TfOz5 into a monophyletic cluster, indicative of high genomic similarity. TfOz5 clusters most closely with the Brazilian K strain, and this is consistent with a relatively recent shared ancestry between TfOz5 and the K strain, based on the species tree topology and extensive orthogroup sharing. This is supported by their tighter grouping in the species tree and extensive orthogroup sharing. Comparative genomic analysis placed TfOz5 within a monophyletic *T. foetus* cluster together with the K and KV-1 strains, supporting its assignment as a geographically distinct Australian representative of this species. TfOz5 shared extensive orthogroup similarity with other *T. foetus* strains, particularly the K strain, while KV-1 showed a lower orthogroup assignment rate. This difference may reflect a combination of biological divergence, differences in gene prediction, assembly structure or annotation strategy rather than true gene content differences alone. Therefore, orthogroup patterns were interpreted cautiously and used primarily to summarize gene family conservation and divergence across the sampled genomes. Across the broader *Parabasalia* comparison, most species showed high orthogroup assignment, indicating a conserved gene-family core among the sampled trichomonads. Species-specific orthogroups were most abundant in *T. musculus* and *T. vaginalis G3*, while TfOz5 contained 305 species-specific orthogroups. These lineage-associated orthogroups provide candidate gene sets for future functional studies, although their biological roles require further validation.

GO enrichment analysis provided additional insight into conserved and divergent functional categories between *T. foetus* strains and between *T. foetus* TfOz5 and *T. vaginalis* G3. Conserved genes between TfOz5 and KV-1 were enriched for functions related to chromosome organization, cell motility, kinase activity and molecular adaptor activity, whereas divergent genes were enriched for terms associated with DNA/RNA catalytic activity and DNA replication. In the comparison between TfOz5 and *T. vaginalis*, conserved genes were associated with core cellular processes, including translation, cell cycle and cytoskeletal organization, while divergent genes were enriched for proteolysis, stress response, transcriptional regulation and host-interaction-related terms. These results highlight candidate functional differences among trichomonads but should be interpreted as hypothesis-generating since GO assignments are based on automated homology-based annotation.

## Conclusion

This study presents a high-quality, chromosome-scale genome assembly of *T. foetus* strain TfOz5, obtained from a naturally infected bull in Queensland, Australia. Using long-read sequencing combined with Pore-C, we achieved significant improvements over previous *T. foetus* assemblies. Comprehensive structural variant analysis, repeat characterization and functional annotations reveal a complex genome architecture rich in repetitive elements and diverse protein-coding genes. Comparative genomics places TfOz5 within the broader *Parabasalia* lineage, highlighting both conserved and lineage-specific gene families. Importantly, this genomic resource opens new avenues for translational research, particularly reverse vaccinology approaches that leverage genomic and proteomic data to identify candidate antigens. Prioritizing surface-expressed or secreted proteins involved in host–pathogen interactions, and integrating transcriptomic and immunological data, will be essential for developing effective recombinant vaccines against bovine trichomonosis.

## Supporting information

10.1017/S0031182026102716.sm001 Santos et al. supplementary material Santos et al. supplementary material

## Data Availability

All raw sequencing reads of the *T. foetus* TfOz5 have been deposited at the National Center for Biotechnology Information (NCBI) under the BioProject accession number PRJNA1432287; BioSample accession number SAMN56340024. The short- and long-read sequences have been deposited in the SRA accessions SRR37642834 and SRR37634590, respectively. The Pore-C sequencing data have been deposited in the SRA accession SRR37634591. The final genome assembly has been deposited in GenBank at JBVWGG000000000.

## Supplementary material

The supplementary material for this article can be found at https://doi.org/10.1017/S0031182026102716.

## Table 1 Long description

Sequencing library rows list platform, number of reads, number of bases, average read length, and Q20 percent. WGS long reads on ONT PromethION report 822,732 reads, 7,770,207,935 bases, average read length 9,444, and Q20 88.43 percent. WGS long reads plus quality filtering report 209,038 reads, 7,336,486,177 bases, average read length 35,097, and Q20 89.65 percent. WGS short reads on Illumina NovaSeq report 55,490,372 reads, 16,647,111,600 bases, average read length 150, and Q20 99.17 percent. WGS short reads plus quality filtering report 55,010,774 reads, 16,393,210,652 bases, average read length 149, and Q20 99.38 percent. Pore-C on ONT PromethION reports 771,212 reads, 438,090,484 bases, average read length 568, and Q20 88.72 percent. Pore-C plus quality filtering reports 757,633 reads, 400,801,765 bases, average read length 529, and Q20 90.12 percent.

Navigate back to Table 1.

## Figure 1 Long description

A circos plot of the TfOz5 genome illustrating multi-layered genomic features across five chromosomes labeled CHR1 to CHR5. The outermost ring represents the chromosomes with tick marks in megabases. The second ring displays gene density as bars, indicating regions of varying gene content. The third ring shows GC skewness as a line, suggesting replication-related patterns. The fourth ring contains dots representing the N-ratio, marking regions with ambiguous base calls. The innermost ring shows sequencing coverage as bars, identifying well-covered regions and potential gaps. Notable patterns include consistently low N-ratio and high coverage on CHR2, indicating fewer assembly uncertainties. Each layer provides insights into different genomic aspects, enhancing the understanding of the TfOz5 genome structure.

Navigate back to Figure 1.

## Figure 2 Long description

Three horizontal bar graphs labeled, BP, MF and CC. BP. Horizontal axis label, Number of sequences. Tick labels, 0, 5000, 10000. Vertical axis categories from top to bottom: viral process; homeostatic process; detoxification; negative regulation of biological process; multicellular organismal process; biological process involved in interspecies interaction; immune system process; response to stimulus; reproductive process; developmental process; localization; regulation of biological process; biological regulation; cellular process. Bar lengths: cellular process is the longest and ends just short of 10000; biological regulation ends a little past 5000; regulation of biological process ends a little past 5000; localization ends between 3000 and 4000; developmental process ends between 3000 and 4000; reproductive process ends between 1000 and 2000; response to stimulus ends between 1000 and 2000; immune system process ends between 500 and 1000; biological process involved in interspecies interaction ends between 500 and 1000; multicellular organismal process ends between 500 and 1000; negative regulation of biological process ends between 0 and 500; detoxification ends between 0 and 500; homeostatic process ends between 0 and 500; viral process ends between 0 and 500. MF. Horizontal axis label, Number of sequences. Tick labels, 0, 2000, 4000, 6000, 8000, 10000. Vertical axis categories from top to bottom: cargo receptor activity; translation factor activity; molecular tag activity; translation regulator activity; general transcription initiation factor activity; antioxidant activity; molecular carrier activity; protein folding chaperone; cytoskeletal motor activity; molecular transducer activity; molecular adaptor activity; structural molecule activity; transcription regulator activity; transporter activity; ATP-dependent activity; molecular function regulator activity; binding; catalytic activity. Bar lengths: catalytic activity is the longest and ends just short of 10000; binding ends between 3000 and 4000; molecular function regulator activity ends between 0 and 500; ATP-dependent activity ends between 0 and 500; transporter activity ends between 0 and 500; transcription regulator activity ends between 0 and 500; structural molecule activity ends between 0 and 500; molecular adaptor activity ends between 0 and 500; molecular transducer activity ends between 0 and 500; cytoskeletal motor activity ends between 0 and 500; protein folding chaperone ends between 0 and 500; molecular carrier activity ends between 0 and 500; antioxidant activity ends between 0 and 500; general transcription initiation factor activity ends between 0 and 500; translation regulator activity ends between 0 and 500; molecular tag activity ends between 0 and 500; translation factor activity ends between 0 and 500; cargo receptor activity ends between 0 and 500. CC. Horizontal axis label, Number of sequences. Tick labels, 0, 3000, 6000, 9000, 12000. Vertical axis categories from top to bottom: protein-containing complex; cellular anatomical structure. Bar lengths: cellular anatomical structure ends just short of 12000; protein-containing complex ends between 0 and 500.

Navigate back to Figure 2.

## Figure 3 Long description

Phylogenetic tree inferred by STAG and rooted by STRIDE, illustrating evolutionary relationships among species such as Trichomonas vaginalis G3 and Tritrichomonas foetus strains. The tree highlights a monophyletic cluster for the T. foetus strains, indicating high genomic similarity. The image B shows a bar chart with the x-axis labeled ′Percentage′ and the y-axis listing species. It measures the percentage of genes assigned to orthogroups, with Trichomonas vaginalis G3 showing the highest percentage near 100 percent, while Histomonas meleagridis has the lowest. The image C shows a bar chart with the x-axis labeled ′Number of Species-Specific Orthogroups′ and the y-axis listing species. Trichomonas vaginalis G3 has the highest count, while Histomonas meleagridis has the lowest. The image D shows a heatmap matrix with species names on both axes, representing pairwise similarities. The color scale ranges from 0 to 2.0 times 10 superscript 4, with darker colors indicating higher similarity. Trichomonas foetus strains show strong similarity, while Histomonas meleagridis shows lower similarity with others. This figure collectively relates phylogenetic data to orthogroup distribution and similarity across species.

Navigate back to Figure 3.

## Figure 4 Long description

Panel A sits at the top left and panel B at the top right, each holding two scatter plots side by side under headers reading conserved on the left and divergent on the right. In every plot the horizontal axis carries log base 10 of observed over expected and the vertical axis carries minus log base 10 of the adjusted p value. Points cluster densely low and to the left, coloured teal green, blue and red, with a few outliers standing higher up the vertical axis and picked out by leader lines running to shaded callout boxes tinted to match the point colour. Panel C fills the lower half as a two column table headed GO term and Definition, holding twenty rows beneath a grey header. The first two rows are shaded red, the next seven blue, and the remaining eleven pale mint, so row colour rather than a separate column carries the category. Definitions run from condensin complex and nucleus through catalytic activities to cell motility and oxidative phosphorylation.

Navigate back to Figure 4.

## Table 2 Long description

Metrics compare genome assemblies across nine Parabasalia genomes, listing assembly level, accession, sequencing technology, size, assembly continuity, gaps, GC content, coverage, and completeness. Assembly level is chromosome for T. foetus TfOz5, T. foetus KV 1, and T. vaginalis G3; scaffold for T. foetus K and H. meleagridis 2922-C6/04 10x; contig for T. foetus Belfast, T. tenax Hs 4:NIH, T. gallinae TGAL, and T. musculus EAF2021. Sequencing technology includes Oxford Nanopore with Illumina and Pore C for TfOz5, Oxford Nanopore with Illumina and Hi C for KV 1, PacBio with Illumina and Hi C for G3, Illumina only for T. foetus K, Oxford Nanopore with Illumina for H. meleagridis, PacBio for Belfast, Illumina for T. tenax and T. gallinae, and PacBio with Oxford Nanopore for T. musculus. Genome size values are 137.7 Mb, 148.7 Mb, 181 Mb, 67.6 Mb, 43.4 Mb, 147 Mb, 63.4 Mb, 55.7 Mb, and 193.5 Mb, in the same column order. Chromosome counts are 5 for TfOz5 and KV 1, and 6 for G3; other columns have no chromosome value. Scaffold counts are 58, 578, 218, 1,479, and 187 for the first five columns, with scaffold N50 values of 24 Mb, 22 Mb, 27.7 Mb, 86.5 kb, and 673.5 kb, and scaffold L50 values of 3, 3, 3, 243, and 16. Contigs are 194, 693, 858, 3,298, 188, 2,776, 4,904, 8,276, and 756, with contig N50 values of 2 Mb, 2 Mb, 541.2 kb, 34.8 kb, 673.5 kb, 82.2 kb, 38.4 kb, 28.1 kb, and 3.6 Mb. Gaps percent are 0.003, 0.01, 0.02, and 3.38 for the first four columns, with no gaps value shown for the remaining columns; GC percent ranges from 28.5 to 33.9 across genomes. Genome coverage values are 77.8x, 76x, 180x, 73x, 200x, 60x, 50x, 100x, and 26x, and genome completeness percent values are 52.9, 52.6, 51.8, 50.6, 52.1, 42.7, 49.8, 48.7, and 53.4.

Navigate back to Table 2.

## Table 3 Long description

Repeat elements are listed with number of elements, total length in base pairs, and proportion in genome percent for TfOz5 and KV 1. Retroelements total 564 elements, 691,637 base pairs, 0.52 percent in TfOz5 and 625 elements, 4,238,700 base pairs, 2.85 percent in KV 1; within these, SINE are 159, 71,806, 0.05 in TfOz5 and 118, 19,701, 0.76 in KV 1, Penelope are 92, 25,866, 0.02 in TfOz5 and not reported for KV 1, LINE are 181, 419,782, 0.31 in TfOz5 and 101, 330,525, 0.22 in KV 1, and LTR elements are 224, 200,049, 0.15 in TfOz5 and 1,714, 777,456, 0.52 in KV 1. DNA transposons are 12,373 elements, 33,002,701 base pairs, 24.64 percent in TfOz5 and 20,695 elements, 48,379,606 base pairs, 32.53 percent in KV 1. Rolling circles are zero for both genomes in number, length, and proportion. Unclassified repeats are 33,010 elements, 24,715,458 base pairs, 18.45 percent in TfOz5 and 33,697 elements, 25,079,482 base pairs, 16.86 percent in KV 1. Total interspersed repeats list 58,435,662 base pairs and 43.62 percent for TfOz5 and 74,586,770 base pairs and 50.15 percent for KV 1, with number of elements not provided. Small RNA are 159 elements, 71,806 base pairs, 0.05 percent in TfOz5 and zero elements, zero base pairs, zero percent in KV 1. Under tandem repeats, simple repeats are 6,328 elements, 320,262 base pairs, 0.24 percent in TfOz5 and 23,238 elements, 1,214,291 base pairs, 0.82 percent in KV 1, and low complexity is 1,721 elements, 133,120 base pairs, 0.10 percent in TfOz5 and 5,875 elements, 354,663 base pairs, 0.24 percent in KV 1.

Navigate back to Table 3.
