# Supplementary material for: Chromosome‑scale genome assembly of the bovine parasite Tritrichomonas foetus using short-read, long‑read and Pore‑C sequencing identifies conserved and lineage‑specific gene families relevant to host interaction
Source: Parasitology. 2026 Aug 11;153(9):1189–205. doi: 10.1017/S0031182026102716 (PMC13735942; doi:10.1017/S0031182026102716)
Supplement: Supplementary file 1 [file S0031182026102716sup001.zip › Supplementary tables S1-S2.docx]

**Table S1** . Antimicrobials used to treat the heavily contaminated field *Tritrichomonas foetus* isolate cultures.

| **Antimicrobial** | **Target** | **Supplier** | **Maintenance concentration** | **Range of concentrations used** |
| --- | --- | --- | --- | --- |
| Amphotericin B | Antifungal | ThermoFisher | 1.2 µg/mL | 0.6 - 4.8 µg/mL |
| Penicillin | Antibacterial | Sigma-Aldrich | 1040 µg/mL | 895 - 1913 µg/mL |
| Streptomycin | Antibacterial | Sigma-Aldrich | 562 µg/mL | 322 - 2002 µg/mL |
| Gentamicin | Antibacterial | ThermoFisher | 16 µg/mL | 8 - 256 µg/mL |
| Tetracycline | Antibacterial | ThermoFisher | 2.5 µg/mL | 2.5 – 12.5 µg/mL |
| Normacin | Broad range:  Fungal  Bacterial  Mycoplasma | InvivoGen | 200 µg/mL | 100 – 400 µg/mL |
| Nystatin | Antifungal | ThermoFisher | 20 µg/mL | 20 – 40 µg/mL |

**Table S2.** Summary of Pore-C run for *Tritrichomonas foetus* TfOz5

|  |  | **Tritrichomonas foetus TfOz5 assembly** |
| --- | --- | --- |
| **Contacts** | Counts (thousand reads) | 771,212 |
|  | Cis**^A^** (%) | 56.9 |
|  | Trans**^B^** (%) | 43.1 |
| **Read length N50 (bp)** |  | 2,633 |
| **Contact order distributions^C^** | 2 (%) | 49.6 |
|  | 3 (%) | 20.3 |
|  | 4-6 (%) | 22.6 |
|  | 7-21 (%) | 7.1 |
|  | 22-50 (%) | 0.39 |
|  | > 50 (%) | 0.01 |

**^A^**Interactions between loci located in the same chromosome (*cis* interactions).

**^B^**Interactions between loci located on different chromosomes (*trans* interactions).

**^C^**Contact order is the number of genome-matched fragments on a single Pore-C read
