# Supplementary material for: Chromosome‑scale genome assembly of the bovine parasite Tritrichomonas foetus using short-read, long‑read and Pore‑C sequencing identifies conserved and lineage‑specific gene families relevant to host interaction
Source: Parasitology. 2026 Aug 11;153(9):1189–205. doi: 10.1017/S0031182026102716 (PMC13735942; doi:10.1017/S0031182026102716)
Supplement: Supplementary file 1 [file S0031182026102716sup001.zip › Supplementary figures.docx]

**Supplementary figures**

**Figure S1**. Schematic representation of *Tritrichomonas foetus* TfOz5 genome statistics. A) GenomeScope profile of the *T foetus* TfOz5 genome based on k-mer frequency analysis from raw sequencing data. B) Contact matrix of the *T. foetus* TfOz5 genome generated using Pore-C data.

**Figure S2.** Linear feature map of *Tritrichomonas foetus* TfOz5 genome. Comprehensive linear representation of the TfOz5 genome, organised into five chromosomes. Each chromosome is displayed as a horizontal track, annotated with key genomic features: genes (blue), coding sequences (CDS; red), introns (orange), start codons (red), and transcripts (green). The physical positions of these features are mapped along the chromosome axes (in megabases, Mb), providing a clear overview of their distribution and density. Feature counts and cumulative lengths are summarised alongside each chromosome, offering insights into genome organisation.
